# Phylogenetic Analysis of H7N9 Avian Influenza Virus Based on a Novel Mathematical Descriptor

**DOI:** 10.1155/2014/519787

**Published:** 2014-06-16

**Authors:** Yusheng Bai, Tingting Ma, Yuhua Yao, Qi Dai, Ping-an He

**Affiliations:** ^1^School of Science, Zhejiang Sci-Tech University, Hangzhou 310018, China; ^2^School of Life Science, Zhejiang Sci-Tech University, Hangzhou 310018, China

## Abstract

A new mathematical descriptor was proposed based on 3D graphical representation. Using the method, we construct the phylogenetic trees of nine proteins of H7N9 influenza virus to analyze the originated source of H7N9. The results show that the evolution route of H7N9 avian influenza is from America through Europe to Asia. Furthermore, two samples collected from environment in Nanjing and Zhejiang and one sample collected from chicken are the sources of H7N9 influenza virus that infected human in China.

## 1. Introduction

In February 2013, two patients with severe pneumonia were admitted to Shanghai Fifth Hospital affiliated with Fudan University [1]. Both patients died in one week, respectively. Chinese experts concluded that the patients were infected with novel avian influenza A H7N9 [2, 3]. 131 people were diagnosed with novel avian influenza A H7N9 in china according to National Population and Family Planning Commission of China, updated to May 7, 2013. This particular A (H7N9) virus had not previously been seen in either animals or people until 2013 in China [4].

The influenza virus consists of eight negative-strand RNA molecules surrounded by an envelope. The envelope contains the HA and NA proteins. There are 17 known HA subtypes and 10 known NA subtypes. Many different combinations of HA and NA proteins are possible. Six of the eight mRNAs code for single proteins, while the remaining two code for two proteins by differential splicing of the RNA. Each mRNA segment is associated with multiple copies of the nucleocapsid protein (NP) and an RNA polymerase (made from the viral proteins PB1, PB2, and PA). H7N9 avian influenza virus is previously only isolated from birds. No case of human infection has been reported before. The Lancet published a research report, in which the H7N9 avian influenza virus has some types of mutations associated with circulation in humans [5].

Graphical representation of biological sequences is one of the most commonly used models to analyze protein sequences [6–26]. Since Hamori and Ruskin proposed H-curve for studying genomic data in 1983, more and more improved graphical representations of DNA sequences were introduced to analyze gene data [6–9]. Then, a number of the graphical representations of DNA sequences were expanded into protein sequences to describe and analyze protein sequences [9–26]. For instance, analogously to the scheme of Jeffrey for graphical representation of DNA, Randić et al. [11, 12], Yang et al. [13], Rasouli et al. [14], and He et al. [15, 16] have suggested several 2D graphical representations of protein sequences. According to the indices of some physicochemical properties of the twenty amino acids, some graphical representations of protein sequences have been proposed to compare the similarities/dissimilarities of proteins [17–26]. Among these graphical representations, 20 amino acids are usually first represented by 20 pregiven vectors. Based on these vectors, an iterated function system is given to generate a curve representing protein sequences. The numerical characterizations of the curves are used to describe corresponding protein sequences. The representation above provides a simple way of viewing, sorting, and comparing various protein sequences.

In this paper, we divided 20 amino acids into five groups based on the two kinds of physicochemical properties of amino acids: charge and polarity. According to the classification, we proposed a novel 3D graphical representation to describe protein sequences. A novel numerical characterization method was suggested, in which each protein sequence can be transformed into eleven vectors. The phylogenetic tree of nine ND5 proteins was constructed to illustrate our approach. The result shows that our method has better representation accuracy and improvement on generation of phylogenetic tree for protein. And then, the phylogenetic trees of nine kinds of protein sequence of H7N9 avian influenza virus were constructed based on this method. The results show that the evolution route of H7N9 avian influenza virus is from America through Europe to Asia. Moreover, the H7N9 influenza virus that infected humans is originated from three strains of which one extracted from chicken in Zhejiang and the others extracted from environment in Nanjing and Zhejiang. Furthermore, all these three strains are originated from the wild bird in Korea which is the earlier source.

## 2. Materials and Methods

### 2.1. Materials

For our analysis, we collected complete protein sequences (HA, M1, M2, NP, NS1, NS2, PA, PB1, and PB2) of the H7N9 influenza virus from NCBI-Flu database with release date updates to June 14, 2013. The number of NA, PA-X, NEP, PB1-N40, and PB1-F2 protein sequences of the H7N9 influenza virus is too small to analyze phylogeny of H7N9 influenza virus. Thus, we took 26 HA protein sequences, 27 M1 protein sequences, 25 M2 protein sequences, 27 NP protein sequences, 28 NS1 protein sequences, 25 NS2 protein sequences, 24 PA protein sequences, 21 PB1 protein sequences, and 21 PB2 protein sequences to construct the phylogenetic tree for H7N9 avian influenza virus. These proteins come from 30 H7N9 avian influenza virus strains around the world.

### 2.2. Methods

#### 2.2.1. 3D Graphical Representation

Amino acids are the structural units that make up proteins. The physicochemical properties of amino acids in a protein are very important factors for three-dimensional structure and chemical reactivity of protein. In the section, we consider two physicochemical properties of amino acids: polarity and charge.

In the work of Huang et al. [23], 20 amino acids were divided into five groups according to polarity and charge: I = {A, V, L, I}, II = {F, W, M, P}, III = {G, S, T, C}, IV = {Y, N, Q, H}, and V = {K, R, D, E}. Both the first sort and second sort of amino acids are nonpolar amino acids, in which the first sort of amino acids is nonpolar aliphatic amino acids and the second sort is nonpolar aromatic amino acids. The third sort and the fourth sort of amino acids belong to uncharged amino acids. The fifth sort of amino acids is composed of positive charged amino acids (lysine and arginine) and negative charged amino acids (aspartic and glutamic).

In this paper, we also adopt the above classification for 20 amino acids. Given a protein sequence *S* = *S*_(1)_ *S*_(2)_ ⋯ *S*_(*n*)_with the length *n*, *S*_(*i*)_ means the *i*th amino acids of the protein sequence; inspect it by stepping one amino acid at a time. Using the classification above, the protein sequence is transformed into *n* points in 2D space by (1)

(1) $$\begin{array}{c} x_{k}=x_{k-1}+a,\;k=2,3,\ldots ,n,\;\;x_{1}=1,\\ y_{k}=b\cdot j+c,\;S_{\left(k\right)}\;\;\text{belong}\;\;\text{to}\;\;j\text{th}\;\;\text{set}. \end{array}$$

For (1), the parameters *a*, *b*, and *c* are arbitrary constants. In the paper, we take *a* = 3, *b* = 10, and *c* = −30. We call the graph as the PC graph of *S*, which is denoted as *C*_*s*_.

For *C*_*s*_, we take the points on the line *y* = 20 as a subsequence *S* ^(1)^ = *S*_(1)_^(1)^ *S*_(2)_^(1)^ ⋯ *S*_(*k*_1_)_^(1)^ of the protein. For the sequence *S* ^(1)^, a map *ϕ*_1_: A → (1,1), V → (−1,1), I → (−1, −1), L → (1, −1) is adopted. Inspecting *S* ^(1)^ by stepping one amino acid at a time, for step *k*  (*k* = 1, 2,…, *k*_1_), the vertex *P*_*i*_ can be constructed according to iterated function system $\vec{X_{i}}=(1/2){\vec{X}}_{i-1}+(1/2)S_{\left(k\right)}^{\left(1\right)}$, where ${\vec{X}}_{i}$ denotes the vector corresponding to the *i*th point. Connecting the adjacent points, a curve can be obtained in 2D space for protein sequence *S* ^(1)^, called *C*_*S*^(1)^_. Then, we construct the L/L matrix to describe numerically the curve *C*_*S*^(1)^_. The elements of the L/L matrix are defined as the quotient of the Euclidean distance between a pair of vertexes of the curve *C*_*S*^(1)^_ and the sum of distances between the same pair of vertexes measured along the curve *C*_*S*^(1)^_. The leading eigenvalue of the L/L matrix is computed, which is denoted as *z* ^(1)^.

Again, when the points are located at line *y* = 10, 0, −10, and −20, operating the same steps, we can also obtain other four parameters *z* ^(2)^, *z* ^(3)^, *z* ^(4)^, and *z* ^(5)^, respectively.

Finally, we construct a 3D graphical representation of protein sequence *S* based on the graph *C*_*s*_ and *z* ^(*i*)^ (*i* = 1,2, 3,4 and 5) as follows:

(2) $$\begin{array}{c} x_{k}=x_{k-1}+3,\;k=2,3,\ldots ,n,\;\;x_{1}=1,\\ y_{k}=10j-30,\;S_{\left(k\right)}\;\;\text{belong}\;\;\text{to}\;\;j\text{th}\;\;\text{set},\\ z_{k}=\overset{5}{\underset{j=1}{\sum }}p_{k}^{\left(j\right)}z_{k}^{\left(j\right)}, \end{array}$$

where *p*_*k*_^(*j*)^ is the quotient of the length of subsequence *S* ^(1)^ and protein sequence *S* with the length *k*. Connecting the adjacent points, we can obtain a curve *G*_*S*_ in 3D space for protein sequence *S*. The 3D graphical representation can be proved to be acyclic and nondegenerate in mathematics. Therefore, the correspondence between protein sequences and graphical curves is one to one.

## 3. Numerical Characterization

In order to numerically characterize a protein, a novel sequence descriptor is introduced as follows.

For *G*_*S*_, we calculate the coordinate of the center point of all points on each line *y* = 20(*l*_1_), *y* = 10(*l*_2_), *y* = 0(*l*_3_), *y* = −10(*l*_4_), and *y* = −20(*l*_5_), respectively, which is denoted as $\left(\bar{x_{k}},\bar{y_{k}},\bar{z_{k}}\right)$ (*k* = 1,2, 3,4, 5) correspondingly to five lines. For the coordinate of the center point of all points on five lines *l*_1_–*l*_5_ in *G*_*S*_, we denote it as $\left(\bar{x_{6}},\bar{y_{6}},\bar{z_{6}}\right)$. Similarly, $\left(\bar{x_{7}},\bar{y_{7}},\bar{z_{7}}\right)$ denotes the center point of all points on four lines *l*_1_–*l*_4_, $\left(\bar{x_{8}},\bar{y_{8}},\bar{z_{8}}\right)$ the center point of all points on three lines *l*_3_–*l*_5_, and $\left(\bar{x_{9}},\bar{y_{9}},\bar{z_{9}}\right)$ the center point of all points on two lines *l*_1_-*l*_2_, respectively. For the points on the line *l*_5_, we divide them into two parts according to the charge, positive or negative. One contains {K, R}; the other contains {D, E}. Using the same calculation above, two center points are obtained, $\left(\bar{x_{10}},\bar{y_{10}},\bar{z_{10}}\right)$ and $\left(\bar{x_{11}},\bar{y_{11}},\bar{z_{11}}\right)$.

Thus, protein sequences can be described by 11 vectors, $\left(\bar{x_{i}},\bar{y_{i}},\bar{z_{i}}\right)$ (*i* = 1–11). For two protein sequences *S* ^*i*^ and *S* ^*j*^, we define their distance as the following equation:

(3) $$\begin{array}{c} D_{ij}=\overset{11}{\underset{k=1}{\sum }}\sqrt{\left({\left(\bar{x_{k}^{i}}-\bar{x_{k}^{j}}\right)}^{2}+{\left(\bar{y_{k}^{i}}-\bar{y_{k}^{j}}\right)}^{2}+{\left(\bar{z_{k}^{i}}-\bar{z_{k}^{j}}\right)}^{2}\right)}. \end{array}$$

The smaller the distance is, the closer the two protein sequences are.

### 3.1. Similarities/Dissimilarities of 9 ND5 Proteins

As discussed above, the similarity of sequences can be compared with the distance among them. To illustrate our method, we consider the numerical characterization of mutations and analyze the similarities among sequences belonging to nine ND5 proteins: human (*Homo sapiens*, P03915), gorilla (*Gorilla gorilla*, P03917), common chimpanzee (*Pan troglodytes*, Q35648), pygmy chimpanzee (*Pan paniscus*, P03916), fin whale (*Balaenoptera physalus*, P24978), blue whale (*Balaenoptera musculus*, P41299), rat (*Rattus norvegicus*, P11661), mouse (*Mus musculus*, P03921), and opossum (*Didelphis virginiana*, P41309), whose sequence data were all downloaded from UniProtKB. The distances among proteins were calculated using (3). If the total number of proteins is *N*, a real symmetric *N* × *N* distance matrix *D* is constructed, whose element *D*_*ij*_ is used to reveal the evolutionary distance between protein sequences *S* ^*i*^ and *S* ^*j*^.

**Figure 1:**
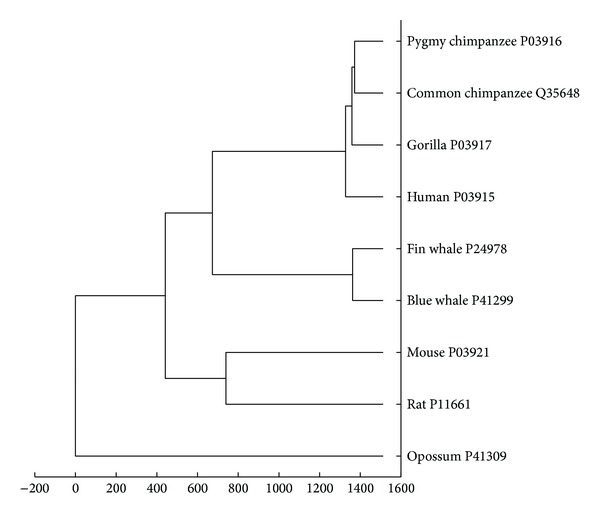
Phylogenetic tree for ND5 proteins of nine species.

Using the UPGMA method [27], the phylogenetic tree is obtained based on the distances between each pair of ND5 proteins, shown in Figure 1, which is consistent with the results obtained with ClustalW methods and some other methods proposed recently [19–23]. The results show that our approach has better representation accuracy and improvement on generation of phylogenetic tree for protein.

## 4. Result and Discussion

**Figure 2:**
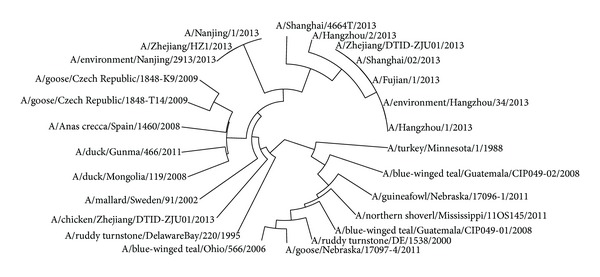
Phylogenetic trees of HA protein of H7N9.

**Figure 3:**
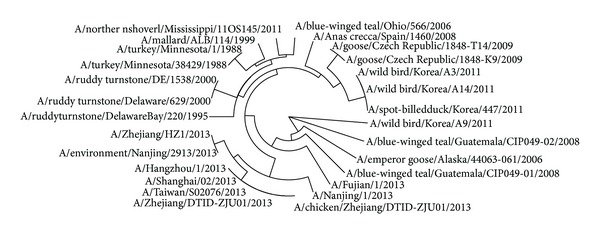
Phylogenetic trees of NP protein of H7N9.

**Figure 4:**
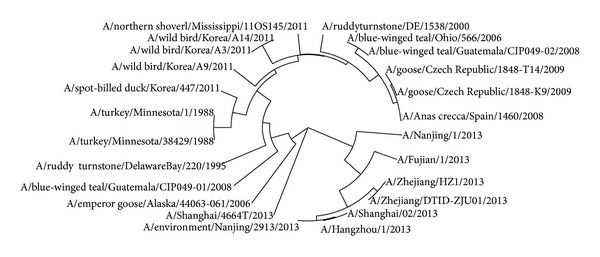
Phylogenetic trees of PA protein of H7N9.

**Figure 5:**
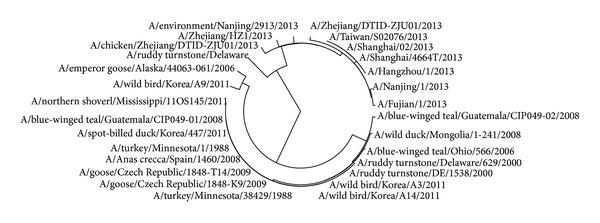
Phylogenetic trees of NS1 protein of H7N9.

**Figure 6:**
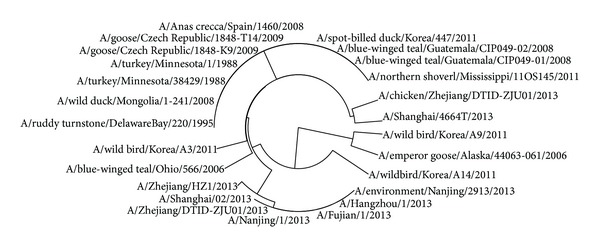
Phylogenetic trees of NS2 protein of H7N9.

**Figure 7:**
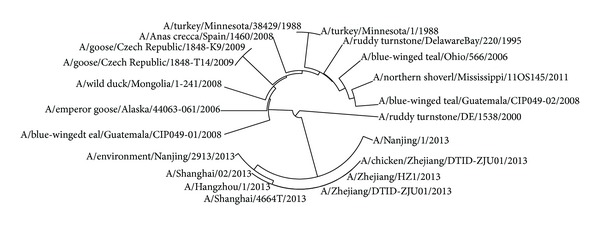
Phylogenetic trees of PB1 protein of H7N9.

**Figure 8:**
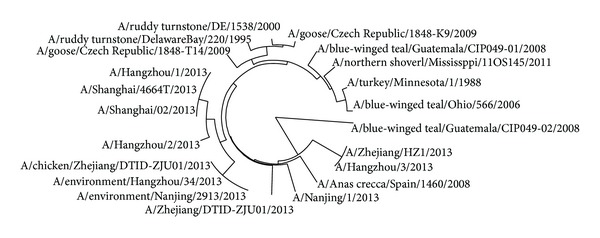
Phylogenetic trees of PB2 protein of H7N9.

**Figure 9:**
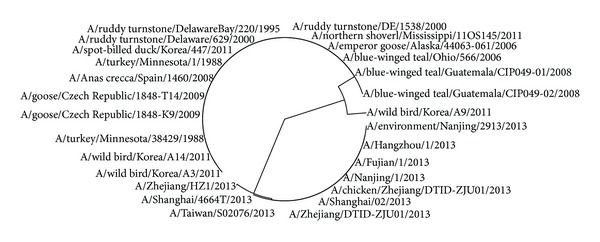
Phylogenetic trees of M1 protein of H7N9.

**Figure 10:**
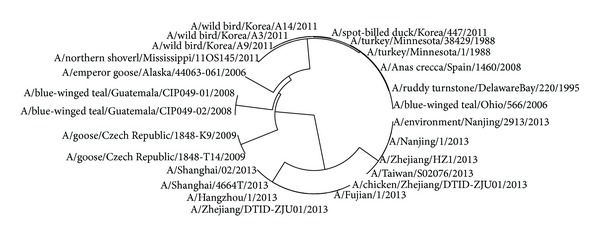
Phylogenetic trees of M2 protein of H7N9.

The avian influenza A virus genome is composed of eight single (nonpaired) RNA strands that can code for up to 14 proteins. For H7N9 influenza virus, we can compare the similarities of nine kinds of protein sequences based on their distances in the section. Using UPGMA method, the phylogenetic tree was obtained, shown in Figures 2–10. The phylogenetic trees of H7N9 genes are consistent with other published studies [4, 5, 28, 29].

Figure 2 shows that the virus strains isolated from the humans are grouped together in one clade. The virus strains, A/Nanjing/1/2013, A/Zhejiang/HZ1/2013, and A/environment/Nanjing/2913/2013, cluster together, which implies the HA protein of virus strains A/Nanjing/1/2013 and A/Zhejiang/HZ1/2013 probably originates from the virus strain A/environment/Nanjing/2913/2013. It is the same with all virus strains in the other cluster and the strain A/environment/Hangzhou/34/2013 is probably the source of the HA protein of all virus strains in the other cluster. Moreover, the evolution route of 26 strains of H7N9 influenza virus suggests the HA protein of virus strain A/chicken/Zhejiang/DTID-ZJU01/2013 most probably originates from America.

In Figure 3, 9 virus strains isolated from the humans are also grouped together in a clade which contains two clusters. One is the virus strains A/Nanjing/1/2013, A/Zhejiang/HZ1/2013, and A/environment/Nanjing/2913/2013, which may show that virus strain A/environment/Nanjing/2913/2013 is the root of others. The other clade contains 5 virus strains, A/Hangzhou/1/2013, A/Shanghai/02/2013, A/Taiwan/so2076/2013, A/Zhejiang/DTID-ZJU01/2013, and A/chicken/Zhejiang/DTID-ZJU01/2013, which indicates that the strain A/chicken/Zhejiang/DTID-ZJU01/2013 is the source of NP protein in this clade.

Similar to Figure 3, the PA proteins of 7 virus strains that infected humans are grouped together in a clade in Figure 4, which suggests that they originate from the strain A/environment/Nanjing/2913/2013. The evolution route of all 24 strains of H7N9 influenza virus suggests America is the earliest source. The complete PA sequences of A/chicken/Zhejiang/DTID-ZJU01/2013 and A/environment/Hangzhou/34/2013 are not provided in the NCBI-Flu database.

In Figure 5, the evolution route of 28 strains of viruses suggests that the strain A/environment/Nanjing/2913/2013 is the source of NS1 protein isolated from humans, whose earlier source is the strain A/emperor goose/Alaska/44063-061/2006 or A/wild bird/Korea/A9/2011. China, Korea, and Alaska are about thousands of kilometers of distance away from each other and human beings could be infected and some of patients contacted poultry before [28]. It may indicate that the strains are sourced probably through poultry trade between the above places.

In Figure 6, the phylogenetic tree of NS2 protein is similar to that of NS1. The strain A/Shanghai/4664T/2013 originates from A/chicken/Zhejiang/DTID-ZJU01/2013 and the strain A/environment/Nanjing/2913/2013 is the source of another NS2 protein of H7N9 virus extracted from human.

Observing the group of virus strains extracted from human in Figure 7, we can see that the virus strain A/chicken/Zhejiang/DTID-ZJU01/2013 is most probably the source of PB1 protein of H7N9 virus extracted from human.

In Figure 8, the PB2 protein sequences of three H7N9 virus strains A/chicken/Zhejiang/DTID-ZJU01/2013, A/environment/Nanjing/2913/2013, and A/environment/Hangzhou/34/2013 are identical, which implies their source should be the same virus. Observing Figure 8, we can see that A/*Anas crecca*/Spain/1400/2008 is the source of PB2 protein of H7N9 virus extracted from human.

All M1 protein sequences of H7N9 virus extracted from China are identical except A/Zhejiang/HZ1/2013, while the other avian H7N9 viruses also possess identical M1 protein sequences except A/Wild bird/Korea/A9/2011 as shown in Figure 9.

There are also two main branches roughly for Figure 10 according to their host. The strains A/environment/Nanjing/2913/2013 and A/chicken/Zhejiang/DTID-ZJU01/2013 are two resources of M2 protein of H7N9 virus extracted from human.

In summary, three strains, A/environment/Nanjing/2913/2013, A/chicken/Zhejiang/DTID-ZJU01/2013, and A/environment/Hangzhou/34/2013, are three main sources of H7N9 virus that infected humans. The phylogenetic trees of PB2 and M1 show that they have identical amino acid sequences. We conclude that three strains originated from the same source.

**Figure 11:**
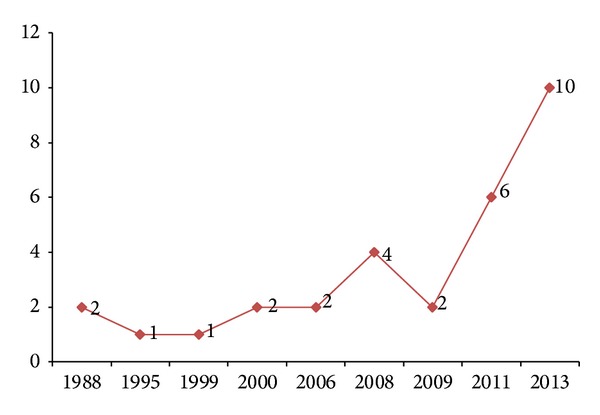
H7N9 avian influenza virus outbreak timeline.

In addition, the H7N9 outbreak at the years 1988, 1995, 1999, 2000, 2006, 2008, 2009, 2011, and 2013 is shown in Figure 11 according to records available in the NCBI-Flu database. The time interval between two outbreaks is 7, 4, 1, and 6 years before the year 2006 and every two years afterwards. The outbreak of H7N9 is more and more frequent, which implies that H7N9 evolution is speeding up.

Observing each distance matrix *D*, we can see the same protein sequences are identical. For instance, we find that the strains A/Fujian/1/2013, A/Shanghai/02/2013, and A/Zhejiang/DTID-ZJU01/2013 have identical HA protein sequences, and the HA protein of strain A/Nanjing/1/2013 duplicated in strains A/Zhejiang/HZ1/2013 and A/environment/Nanjing/2913/2013. All identical protein sequences are listed in Table 1.

**Figure 12:**
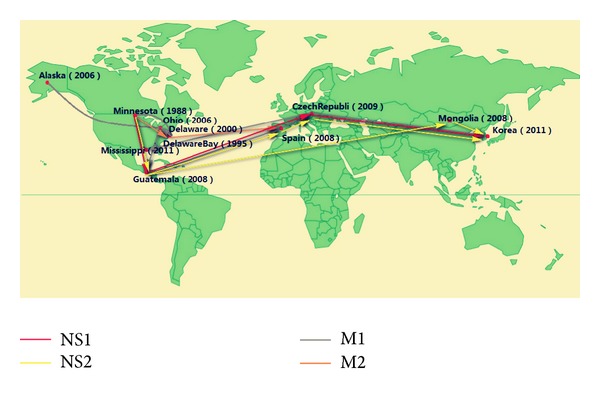
Location of identical M1, M2, NS1, and NS2 sequences found widely separated in space and time for H7N9 avian influenza virus.

From the perspective of geography [30], we show identical sequences (NS1, NS2, M1, and M2) occurring in different locations connected by arrow lines in Figure 12. Observing the date and location of occurrence for each identical protein sequence above, we find that the earliest H7N9 avian influenza virus comes from America, spreading to Europe and finally arriving in Asia. For example, M1 protein sequence of H7N9 avian influenza started from Minnesota (1988), passing Delaware Bay (1995), Delaware (2000), Alaska (2006), and Ohio (2006), then Guatemala (2008), Spain (2008), and Czech Republic (2009), and finally arrived at Korea in Figure 11 (2011). In another instance, the evolution route of NS1 protein sequences of H7N9 avian influenza virus is also from America (Minnesota, 1988; Guatemala, 2008) through Europe (Czech Republic, 2009) to Asia (Korea, 2011).

Comparing the results of phylogenetic trees of nine kinds of H7N9 protein, we can see that the evolution route of H7N9 avian influenza virus is from America through Europe to Asia.

## 5. Conclusion

The physicochemical properties of amino acids determine three-dimensional structure and the biological activity of the protein. In the paper, according to charge and polarity of 20 amino acids, we divided them into five sorts. Then a novel 3D graphical representation was proposed, called *G*_*S*_ Curve, for a protein sequence. Using the defined distance, we obtain similarity matrix *D* by computing the distance of nine kinds of H7N9 protein sequences. By the single linkage method, their phylogenetic trees are constructed based on similarity matrix *D*. To show the utility of the approach, the phylogenetic tree of nine ND5 proteins was constructed.

Furthermore, the phylogenetic trees of nine kinds of protein sequences of H7N9 avian influenza virus were constructed based on this method. From the phylogenetic trees of nine kinds of H7N9 protein sequences, we deduce that two samples collected from environment in Zhejiang and Nanjing and one sample collected from chicken in Zhejiang are sources of strains that infected humans in 2013. The phylogenetic tree of PB2 and M1 shows that the three strains may originate from the same source. The result is supported by the geographic analysis of virus outbreak record.

## Figures and Tables

**Table 1:** Statistics information of identical protein sequences of H7N9 available in the NCBI-Flu database.

HA	AGK82158-AGL44438-AGJ51953; AGJ73503-AGM16242-AGJ73510;

NP	ACX53684-ADN34728; AGK84853-AGL44439-AGL95089-AGJ51954-AGJ72862; ABI84698-ACZ46730; AEK84689-AEK84687;

PA	ACX53682-ADN34726; AGK84852-AGL44436; AGJ51952-AGM16237; ABI84701-ACZ46350; AEK84707-AEK84708;

NS1	ACX53686-ADN34732-ADN34743-ADK71142-AEK84817-ABI84699-ACZ47493-AEK84809-AEK84811; AGL44443-AGI60296-AGL95093; AAY96591-AAY96593;

NS2	ACX53687-ADN34733-ADN34744-ADK71143-ADK71154-AEK84818-ABI84700-ACZ47494-AEM98235; AGK82163-AGK84854-AGJ73509-AGL44444-AGJ51958-AGJ73516;

PB1	ACX53680-ADN34724; AGK84851-AGI60294-AGJ51960-AGM16239-AGJ72859; ABI84702-ACZ45969;

PB2	AGK84859-AGM16245; AGJ72858-AGK84862-AGJ73521;

M1	AGK82160-AGI60298-AGJ73506-AGL44441-AGI60290-AGL95091-AGJ51957-AGJ72864-AGJ73511; ACU44782-ADN34730-ADN34741-ADK71140-ADK71151-ABS89410-AFM09448-AGE08099-AAY96476-AFX85261-AAY96478-AEK84670-ABI84695-ACZ48319-AEK84654-AEK84656;

M2	ACU44783-ADN34731; ADN34742-AFX85262-ABI84696-ACZ48320; AGK82161-AGI60299-AGL44442-AGL44442-AGI60291-AGJ51956-AGJ72865; AGJ73507-AGL95092-AGM16244-AGJ73512; ADK71141-ADK71152; AEK84671-AEK84655-AEK84657-AEK84659;
